# Spatial measurement errors in the field of spatial epidemiology

**DOI:** 10.1186/s12942-016-0049-5

**Published:** 2016-07-01

**Authors:** Zhijie Zhang, Justin Manjourides, Ted Cohen, Yi Hu, Qingwu Jiang

**Affiliations:** Department of Epidemiology and Biostatistics, School of Public Health, Fudan University, Shanghai, 200032 China; Key Laboratory of Public Health Safety, Ministry of Education, Shanghai, 200032 China; Department of Health Sciences, Northeastern University, Boston, MA 02115 USA; Department of Epidemiology and the Center for Communicable Disease Dynamics, School of Public Health, Harvard University, Boston, MA 02115 USA; Division of Global Health Equity, Brigham and Women’s Hospital, Boston, MA 02115 USA; Harvard Medical School, Boston, MA 02115 USA

**Keywords:** Spatial epidemiology, Environmental epidemiology, GIS, Geographical epidemiology, Measurement error, Misclassification

## Abstract

**Background:**

Spatial epidemiology has been aided by advances in geographic information systems, remote sensing, global positioning systems and the development of new statistical methodologies specifically designed for such data. Given the growing popularity of these studies, we sought to review and analyze the types of spatial measurement errors commonly encountered during spatial epidemiological analysis of spatial data.

**Methods:**

Google Scholar, Medline, and Scopus databases were searched using a broad set of terms for papers indexed by a term indicating location (space or geography or location or position) and measurement error (measurement error or measurement inaccuracy or misclassification or uncertainty): we reviewed all papers appearing before December 20, 2014. These papers and their citations were reviewed to identify the relevance to our review.

**Results:**

We were able to define and classify spatial measurement errors into four groups: (1) pure spatial location measurement errors, including both non-instrumental errors (multiple addresses, geocoding errors, outcome aggregations, and covariate aggregation) and instrumental errors; (2) location-based outcome measurement error (purely outcome measurement errors and missing outcome measurements); (3) location-based covariate measurement errors (address proxies); and (4) Covariate-Outcome spatial misaligned measurement errors. We propose how these four classes of errors can be unified within an integrated theoretical model and possible solutions were discussed.

**Conclusion:**

Spatial measurement errors are ubiquitous threat to the validity of spatial epidemiological studies. We propose a systematic framework for understanding the various mechanisms which generate spatial measurement errors and present practical examples of such errors.

## Background

Studying the distribution of health-related events in specified population, over time and across space is the business of epidemiologists [1]; however, until a decade ago, the use of spatial data was mainly descriptive. As the rapid development of spatial information techniques [e.g., geographic information systems (GIS), remote sensing (RS), and global positioning systems (GPS)], the availability of spatially referenced health data and associated risk factors in digital format has increased greatly [2–4]. This has been accompanied by the appearance of new software packages for spatial data analysis. Together, these developments have created unprecedented new opportunities for researchers to investigate the association of geographically indexed health events with various demographic, environmental, behavioral, socioeconomic, and genetic risk factors to explore and explain geographic variation in disease risk. Researchers are becoming more familiar with space-related epidemiological studies (hereafter referred to as “spatial epidemiology”) [5–7].

As access to spatial data and spatial analytic approaches advances, so does the need to address sources of bias in spatial epidemiology. In most studies of spatial epidemiology, the data are assumed to be reliable and free of measurement errors. But in practice, this is often not the case. Measurement errors can affect the data through several mechanisms and many different stages of data collection and analysis (the term “measurement errors” is most commonly used for continuous variables and “misclassification” for categorical variables, but for convenience we will use the term of measurement errors in the remainder of the paper since misclassification can be considered a special case of measurement errors). Spatial measurement errors can be random (such as errors originating from a GPS device) or there may be quantifiable factors contributing to the errors (such as errors due to imperfect sensitivity or specificity of a diagnostic test, latency periods of diseases, multiple addresses, etc.). While the concept of measurement errors is often discussed in classical epidemiology [8–10], it has not yet received much discussion as it relates to spatial epidemiologic studies [11].

Spatial epidemiologic data is like all epidemiological data, except it has at least one additional attribute describing the spatial or geographical location for each observation. Hence, some measurement errors encountered in spatial epidemiology are analogous to those previously described in classical epidemiology, but the spatial component of the data introduces additional measurement errors which need to be described, categorized, and accounted for Elliot et al. and Beale et al. [3, 4, 11] described this issue with regard to bias and confounding, but there has not been a systematic investigation of location-based measurement errors from the spatial perspective. A rigorous discussion and classification of these types of spatial errors is vital to ensure valid inference can be obtained from spatial epidemiologic studies [4].

## Methods

Searches for relevant studies were carried out using two academic databases (Medline and Scopus) between the available earliest date and December 20, 2014. Databases were searched using a broad search term purposefully relating to “space” (space or geography or location or position) as well as a term relating to “measurement error” (measurement error or measurement inaccuracy or misclassification or uncertainty). The search strategy was (spatial [Title/Abstract] OR space [Title/Abstract] OR geographic * [Title/Abstract] OR geography [Title/Abstract] OR locational [Title/Abstract] OR location [Title/Abstract] OR positional [Title/Abstract] OR position [Title/Abstract]) AND (measurement error * [Title/Abstract] OR measurement inaccurac * [Title/Abstract] OR misclassification * [Title/Abstract] OR uncertaint * [Title/Abstract]) AND (english [Language]). We manually reviewed the abstracts to judge the relevance with spatial measurement errors and then we reviewed the cited references in the selected papers to identify additional potential articles. Those studies focusing on statistical methods or epidemiologic issues were also included in this review, while studies focusing mainly on technical problems such as equipment usage were deemed out of scope for the current review.

### Conceptual framework for classifying sources of spatial measurement errors

**Fig. 1:**
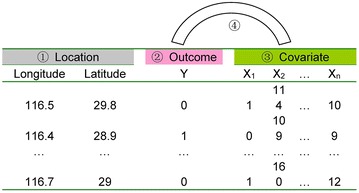
Schematic framework for spatial measure errors in spatial epidemiology. For *Location* (①), the geographic coordinates are used as an example here. In practice, the Cartesian coordinates can be used instead, which is the coordinates used in the process of data analysis; For *Outcome* (②), a dichotomous variable is used for an example and only one column is needed. Other types of *Outcome* variables can also have more than one dimension, such as Poisson data, that may include a numerator (e.g., number of cases) and denominator (population at risk). For simplicity, only one column is used to indicate the *Outcome*. The *Covariates* (③) may be any combination of categorical or continuous variables. The error caused by the correspondence between *Outcome* and *Covariates* is marked as ④

To discuss the spatial measurement errors systematically, we suggest the following conceptual framework as a helpful way to effectively organize the topic. We recognize that there is no way to completely capture the intricacies of all possible routes of measurement errors, however we present a basic structure, that can be expanded upon, to begin the process of systematically categorizing the types and structure of these potential errors. Spatial epidemiologic studies include two additional variables recording the locations/positions of observations in the leftmost two columns, while the other outcome and covariate variables are often similar to those used in classical epidemiology (Fig. 1).

The following four types of spatial measure errors are divided accordingly from the point of application:

1. Pure spatial location measurement errors;
2. Location-based outcome measurement errors;
3. Location-based covariate measurement errors; and;
4. Covariate-Outcome spatial misaligned measurement errors.

### Theoretical model to characterize spatial measurement errors

These sources of spatial measurement errors can be integrated in a mathematical framework. Spatial epidemiology studies include two variables representing outcome locations (L), outcome measurements (Y), and covariate measures (X) that may include spatial information as well. Consider one example where the unit of analysis is the individual, and a researcher is trying to identify associations between cancer status and exposure to contaminated drinking water. In this setting L would represent the individual’s location, Y would be that individuals case or control status, and X could represent an individual’s spatially varying exposure (e.g., amount of exposure to contaminated water). In another setting, one may be interested in relating deaths in a zip code to air pollution concentrations measured at an air pollution monitoring site. In this second scenario, L would be the common location assigned to everyone in the same zip code, Y would be the number of deaths observed, and X would be the spatially varying measure of air pollution that would be assigned to all individuals within a certain radius of the monitoring site. Suppose (*L*_0_, *Y*_0_, *X*_0_) are the true measures of the outcome location, outcome, and covariate measures, respectively. In practice this information is measured with some errors. Let (*L*, *Y*, *X*) are the observed values of the outcome location, outcome, and covariate measures, which are the true value plus measurement errors Δ, Δ = (Δ_L_, Δ_Y_, Δ_X_). Each of these individual measurement errors may be a function of location, outcome, or exposure, or a combination of these measures. We present a simple linear combination framework for these potential errors below, but stress that the relationships between truth and measurements may be more complex. In the situation where more complex relationships may exist, these models can include more advanced modeling terms such as interactions between measurements, polynomials, and indicator random variables, or even distributional assumptions on the multiplicative coefficients. The basic formulation for our measurement error framework is given as follows:

$$ \begin{aligned} L & = L_{0} + \,\Delta _{\text{L}},\quad {\text{ where }}\Delta _{\text{L}} = \gamma_{0} {\text{L}}_{0} + \gamma_{1} {\text{Y}} + \gamma_{2} {\text{X}} + \upvarepsilon_{\text{L}} \\ Y & = Y_{0} + \,\Delta _{\text{Y}} ,\quad {\text{ where }}\Delta _{\text{Y}} = \gamma_{3} {\text{L}} + \gamma_{4} {\text{Y}}_{0} + \gamma_{5} {\text{X}} + \upvarepsilon_{\text{Y}} \\ X & = X_{0} + \,\Delta _{\text{X}} ,\quad {\text{ where }}\Delta _{\text{X}} = \gamma_{6} {\text{L}} + \gamma_{7} {\text{Y}} + \gamma_{8} {\text{X}}_{0} + \upvarepsilon_{\text{X}} \\ \end{aligned} $$

In the above equations, we define ε_L_, ε_Y_, and ε_X_ as the residual measurement errors remaining that is not directly related to the linear combination of the outcome, exposure, or covariates.

## Results

7131 papers in Medline and 7595 papers in Scopus were found, where 149 papers were identified as closely related with the topic discussed in this study and were read thoroughly. 97 literatures were cited here.

### Pure spatial location measurement errors

Pure spatial location measurement errors are introduced by inaccuracies in the positioning of spatial/geographical locations that will affect the outcome and covariate simultaneously. These errors can be broken down to instrumental errors (e.g., global positioning systems errors) and non-instrumental errors (e.g., multiple address, geocoding errors, outcome and covariate aggregations). We demonstrate how even pure spatial location measurement errors can lead to mismeasurement of both the outcomes and covariates, and accordingly, in this situation, the observed data is of the form (*L*, *Y*, *X*), where no *γ*s are guaranteed to be non-zero.

#### Instrumental errors

Instrumental errors are caused by inaccuracies in the tools used to measure the spatial locations (e.g., GPS). It is a worldwide, satellite-based, radio-navigation system developed by the U.S. Department of Defense (DOD) and may be the most widely used tool to obtain the geographical locations in spatial epidemiologic studies. A ground-based GPS receiver calculates the time it takes for individual signals to arrive from at least three satellites to the receiver to compute a two-dimensional location (latitude and longitude), given an assumed height. And the detection of satellite signals of four satellites can determine three-dimensional positions and time, whereas five or more can provide greater precision [12]. The accuracy of these GPS methods have been well reported [13]. It has been shown that positional errors resulting from inaccuracies in GPS system influence spatial analytic methods by inflating standard errors of estimates, which results in reduced power to detect spatial clustering and spatio-temporal trends. For example, Armstrong et al. [14] used the classical North Humberside leukemia and lymphoma case–control data to quantify the effects of increasing levels of positional error perturbation on the deterioration of the power of the Cuzick–Edwards test for spatial clustering; Cassa et al. [15] added artificial clusters of various shapes and sizes to data on residence locations of individuals making hospital emergency department visits for respiratory illness. These locations were then perturbed at various levels according to a bivariate normal distribution with standard deviation inversely proportional to the local population density. The ability of the spatial scan statistic to detect the clusters was quantified and shown to decline as the level of perturbation increased; Olson et al. [16] used essentially the same baseline data as Cassa et al. [15], but moved the locations to zip code or census tract centroids rather than perturbing them according to a normal distribution, and obtained the qualitatively similar results; Gabrosek and Cressie [17] and Cressie and Kornak [18] investigated, via simulation, the impact of circular uniform and normal perturbation of location errors on kriging approach and trend estimation and the latter authors showed how spatial autocorrelation in a geostatistical model attenuates as the perturbation level increases.

However, purely location-based measurement errors are inevitable. For example, GPS accuracy is affected by many factors such as the atmospheric conditions affecting the velocity of GPS signals, obstructions such as concrete and steel within many buildings preventing reception of GPS signals, and multi-path errors arising from the reflection of satellite signals from other surfaces including buildings, vegetation, the ground or water [19]. The actual errors may range from only centimeters to hundreds meters [12, 20]. In practical applications, the main consideration for addressing this type of errors is to balance these instrumental errors with the study scale.

In the presence of these instrumental errors, we observe the outcome (Y) accurately (*γ*_3_ = *γ*_4_ = *γ*_5_ = 0). The errors in the measured locations are not related to the outcome, or the covariates (*γ*_1_ = *γ*_2_ = 0). As some covariates may depend on the mis-measured locations (i.e. PM_2.5_ exposure at an individual’s home address), observed covariates can be biased, meaning non-zero contributions of *γ*_6_ and *γ*_8_. The observed data takes the form (*L*, *Y*_0_, *X*).

#### Non-instrumental errors

##### Multiple addresses

Multiple addresses indicate that the studied cases/individuals have several locations where exposure or onset of disease may have occurred. Accordingly, multiple addresses raise the possibility of positional uncertainty since it may be difficult to ascertain the actual location for the outcome(s) and relevant covariate measurements. At the same time, capturing address history may provide the opportunity to adjust for this uncertainty when attempting to link exposures with outcomes or when seeking to identify spatial clusters of cases.

For example, studies of environmental exposures and adverse birth outcomes often rely on maternal address at birth obtained from the birth certificate to classify exposure. Although the gestational age of interest is often early pregnancy, maternal addresses are not available for women who move during pregnancy when using maternal addresses abstracted from birth certificates. Chen et al. [21] explored the extent of ambient air pollutant exposure measurement error due to maternal residential mobility during pregnancy among a subgroup within a New York birth cohort, but no significant impact was found, which may mainly because of limited population mobility; A study of breast cancer in Upper Cape Cod, Massachusetts demonstrated potential exposure uncertainty introduced by a mobile population [22]. The authors conducted three separate analyses on a sample of breast cancer cases: (1) all cases were considered (2) only cases that have lived at the same location for at least 15 years were considered and (3) only cases that have lived at the same location for at least 20 years were considered. The overall results associating location and disease (across three separate cluster detection techniques) showed decreasing *p* values as the lag increases. This result provides evidence of a spatial association that is stronger when only those cases that have not recently moved are included.

Several other studies have attempted to recognize the influence of multiple addresses, generally by only analyzing residential locations during a pre-specified time interval. One such study found that the most likely cluster of lymphoma in a case–control study was found by examining addresses with a 20 year lag period compared to the 5, 10, 15 years lag period [23]. Han et al. [24] used kernel density estimation methods to identify clustering of breast cancer using residential histories. Sabel et al. [25] examined clustering of Amyotrophic Lateral Sclerosis in Finland based on place of birth and place of death, respectively, showing incomplete agreement. Gallagher et al. [26] used residential histories to assess the affect of drinking water exposure to breast cancer, by examining any previous address where a study participant was exposed to public drinking water impacted by effluent.

Another potential solution for using multiple addresses involves using weighted distance-based methods to account for multiple addresses. By basing tests on the distances between locations of interest, researchers can give increased weight to addresses that may be more important (or informative) and less weight to addresses that may be less important (or less informative). Weighted distance based cluster detection methods have been shown to improve power to detect clustering in mobile populations [27].

In the presence of multiple addresses, independent of the outcome or covariate, we observe the outcome (Y) accurately (*γ*_3_ = *γ*_4_ = *γ*_5_ = 0). The errors at measured locations are not influenced by the outcome, or the covariates (*γ*_1_ = *γ*_2_ = 0). As multiple addresses could affect the measured covariate (as is the case in Gallagher et al.) [26], *γ*_6_ and *γ*_8_ may be non-zero. The observed data takes the form (*L*, *Y*_0_, *X*). If the multiple addresses are not independent of the outcome or covariate (i.e. moving closer to elderly people moving to care facilities, or people moving as a result of a shift in socioeconomic status), then we can no longer assume that the corresponding *γ* terms are zero, and the observed data may take the form (*L*_0_, *Y*_0_, *X*) (*L*, *Y*_0_, *X*_0_), or (*L*_0_, *Y*_0_, *X*_0_).

##### Geocoding errors

Geocoding requires the matching of an address of interest to an address-ranged street segment georeferenced within a street-line database, followed by interpolation of the position of the address along that segment. This is always done through automated geocoding techniques widely used in geographical analyses. Unfortunately, geocoding results obtained by these automated procedures are well-known to contain positional errors of several hundred meters or more (defined here as the (vector) difference between the location of an address ascertained by automated geocoding and its corresponding ground-truth location) [28–41]. For example, in the study on a four-county area of upstate New York, Cayo and Talbot [31] found that 10 % of a sample of rural addresses geocoded with errors of more than 1.5 km, and 5 percent geocoded with errors exceeding 2.8 km; In a case study of Orange County, Florida, Zandbergen and Green [42] determined the effect of positional errors in geocoding on the analysis of exposure to traffic-related air pollution of children at school locations through comparisons with a parcel database and digital orthophotography, suggesting that typical geocoding is insufficient for fine-scale analysis of school locations; Mazumdar et al. [43] found, via simulation, that the magnitude of the odds ratio (OR) measuring the relationship between covariates (environmental exposure) and the outcome (disease) generally declined with decreasing geocoding accuracy; Zimmerman et al. [40] sought to model the probability distribution of positional errors associated with automated geocoding, where the mixtures of bivariate *t* distributions with few components appear to be flexible enough to fit many positional error datasets associated with geocoding.

A special case for geocoding errors is when no locations can be found by geocoding. The standard approach for handling these missing geocodes is to discard those observations and analyze only complete observations. Reich et al. [44] proposed a hierarchical Bayesian spatial model to handle missing observation locations. Through a simulation study, it was found that this method may allow for more reliable epidemiological analysis. The authors also applied this method to a study of the relationship between fine particulate matter and birth outcomes in southeast Georgia. Oliver et al. [45] described geographic bias in GIS analyses with unrepresentative data owing to missing geocodes, using as an example a spatial analysis of prostate cancer incidence among whites and African Americans in Virginia, 1990–1999. They found that cluster maps showed patterns that appeared markedly different, depending upon whether one used all cases or those geocoded to the census tract. Geocoding errors will also result in exposure measurement error, which will depend on the spatial variation of the exposure being studied [35].

In the presence of geocoding errors, the errors in the measured locations are not influenced by the outcome (*γ*_1_ = 0), but may be influenced by certain covariates (i.e. rural vs urban) leading to a potentially nonzero contribution of *γ*_2_. If we consider the outcome (Y) to be a count of the number of cases of a specific disease in a census tract or zip code, then the mismeasurement of locations of cases could influence these values and (Y) may also be inaccurate (*γ*_4_ = *γ*_5_ = 0). Some covariates may still depend on the location (which may be measured with error) resulting in non-zero contribution of *γ*_6_ (*γ*_7_ = *γ*_8_ = 0). The observed data takes the form (*L*, *Y*, *X*).

##### Outcome aggregations

Aggregation is a manipulation of data that is widely used in spatial epidemiologic studies either because of the availability of associated data or the need to protect confidentiality. Aggregation is usually performed at the level of particular administrative units. Upon aggregation of outcomes, researcher will need to specify a location to represent the aggregated outcome for the spatial data analysis. The centroid of the aggregated unit (e.g., postal/zip codes, census tracts, dissemination areas, blocks or block groups) is frequently used as the address proxy for sample unit locations, but this will always serve as a source of errors. Additionally, aggregation masks the original detailed outcome information; this may be even more complicated if the original (i.e. disaggregated) outcome included measurement errors.

Waller and Jacquez [46, 47] demonstrated empirically that the effect of aggregation on tests for focused spatial clustering and space–time clustering reduced power, and that the amount of power reduction was directly related to the level of aggregation. Ozonoff et al. [48] reported that increasing levels of aggregation led the spatial scan statistic to not only lose power to detect disease clusters but also to increase the false detection rate. Berke and Waller [49] used regional aggregated count data to investigate the measurement error effect of West Nile virus infections among dead birds sampled from the 30 public health units of southern Ontario in 2005 on semivariogram, Moran’s *I* statistic and the spatial scan test. They found that no serious spatial bias was introduced by the use of an imperfect diagnostic test as long as the imperfection itself was spatially unbiased.

Aggregation is closely related with the well-known modified area unit problems (MAUPs), which remains an unsolved problem in geography. But if the aggregation bias includes the original outcome measurement errors, then Bayesian approaches with probability formulas and prior information on the potential measurement error probabilities of outcomes may be a promising approach for mitigating this issue [50, 51].

In the presence of outcome aggregations, the errors associated with the measured locations are not influenced by the outcome, or the covariates (*γ*_1_ = *γ*_2_ = 0). But the outcome (Y) is not accurate for either the locations or the measurement (*γ*_5_ = 0), which results in the inaccuracy of some covariates (*γ*_6_ = *γ*_7_ = 0). The observed data takes the form (*L*, *Y*, *X*).

##### Covariate aggregation

Covariate aggregation means that the aggregated covariates were used in place of individual covariates. For example, socio-economic covariates (e.g., gross domestic product, GDP) in aggregated units are sometimes used to represent the socio-economic status of individuals.

The impact of covariate aggregation in classical epidemiological studies has been well described, but in spatial epidemiologic studies the impact of such aggregation has not been explored in great detail. Many studies reviewed have simply combined the covariates at different scales in the process of spatial analysis, ignoring completely the potential effects of covariate aggregation [52–54]. For example, Raso et al. [55] used an integrated approach for risk profiling and spatial prediction of coinfection with Schistosoma mansoni and hookworm for western Côte d’Ivoire through combining demographic, environmental, and socioeconomic data, where the Normalized difference vegetation index and land surface temperature data with a spatial resolution of 1 km and the Rainfall estimate data with an 8 km spatial resolution were used.

Hierarchical modeling and analysis for spatial data, which considers the spatial relationships and hierarchical structures is one promising approach for dealing with covariate aggregation [56]. For example, Yang et al. [57] used a hierarchical multi-level model to explore the risk factors of schistosomiasis japonica by nesting the individual variables such as gender, age and occupation within the village level variables such as type of *S.japonicum* endemic area, drinking water source, and sewage treatment. However, methods to correct the covariate aggregation from different spatial resolutions as described above (i.e. not the obvious different scales such as county, village, individuals) and the associated impacts on the study results remained unexplored.

In the presence of covariate aggregations, the errors in the measured locations are not influenced by the outcome, or the covariates (*γ*_1_ = *γ*_2_ = 0). The covariate (X) is not accurate for either the locations or the measurement (*γ*_7_ = 0), while the outcome (Y) is accurate (*γ*_3_ = *γ*_4_ = *γ*_5_ = 0). The observed data takes the form (*L*, *Y*_0_, *X*).

### Location-based outcome measurement errors

Pure outcome location measurement errors mean that the outcome measurements have errors in some locations (e.g., a case is misclassified as noncase), which will only affect the outcome. It becomes more complicated because of its location attributes compared to non-spatial situations and may include two different types: purely outcome measurement errors, and missing outcome measurements.

In the presence of purely outcome-based measurement errors or missing outcome measurements [58], the errors in the measured locations are assumed to be zero (*γ*_0_ = *γ*_1_ = *γ*_2_ = 0). The outcome (Y) is not accurate for the measurement (*γ*_3_ = *γ*_5_ = 0), while the covariate is accurate (*γ*_6_ = *γ*_7_ = *γ*_8_ = 0). The observed data takes the form (*L*_0_, *Y*, *X*_0_), as there is only mismeasurement of the outcome.

#### Purely outcome measurement errors

**Fig. 2:**
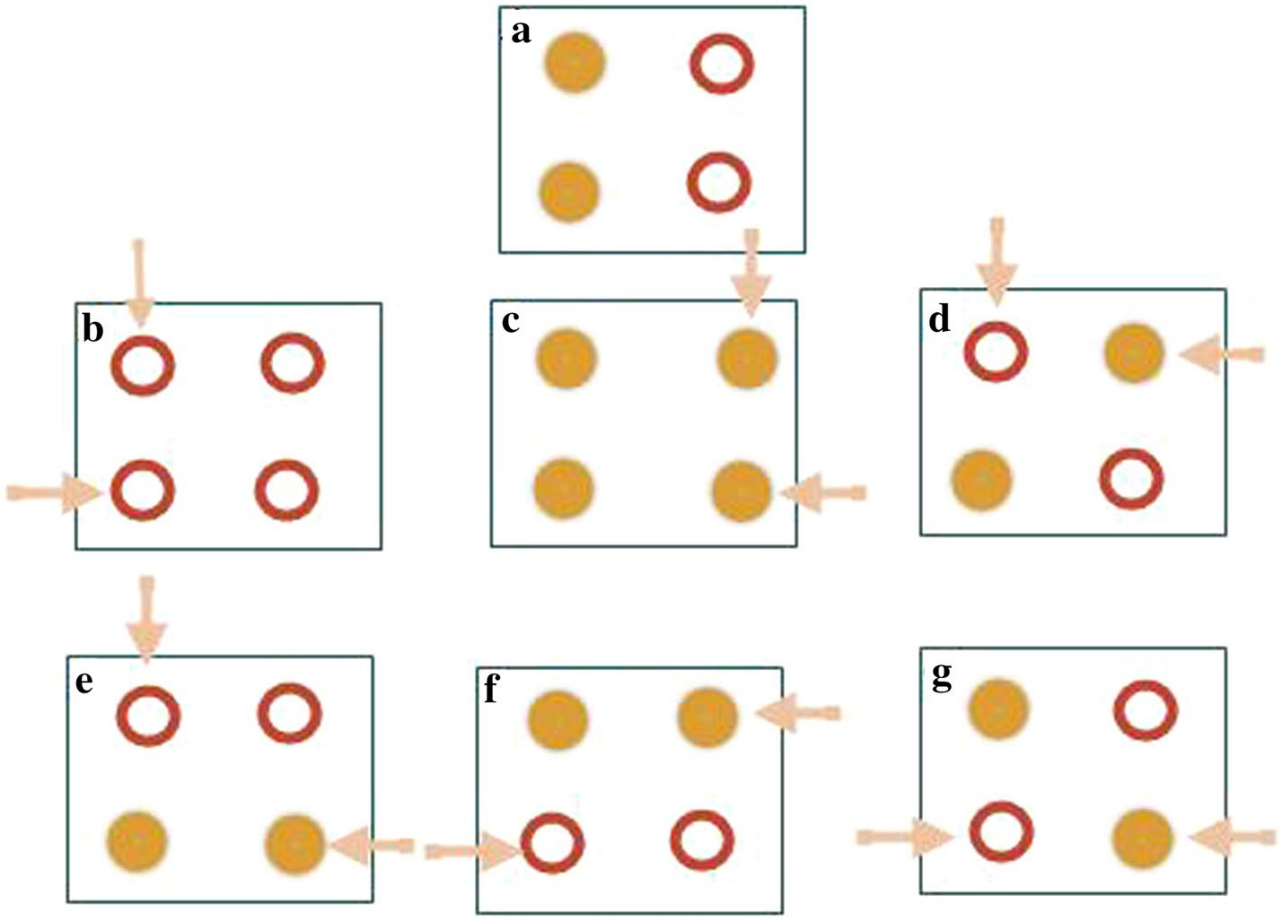
Location-based outcome measurement errors (**a** is the correct pattern and **b**–**g** shows the six possibilities with different outcome measurement errors). *Filled circles* represent cases and *hollow circles* represent controls

In order to describe this form of measurement errors we introduce a simple example with a binary outcome. Suppose there are four locations and the true outcome includes two cases and two controls as shown in Fig. 2a. Say there are two measurement errors. Figure 2b–g shows six possibilities with different spatial patterns: one two-case misclassification, one two-control misclassification, and four one-case and one-control misclassifications.

In schistosomiasis studies, the fecal examination is always used as the “gold standard” test to diagnose a disease. However, as it is more difficult, and potentially more time consuming, to prove the complete absence of eggs in a clean stool sample than it is to prove the presence of eggs in a contaminated sample, especially in the low endemic regions, there could be many false negatives. In this situation, we may expect preferential misclassification of (L, Y_0_ = 1, X) to (L, Y = 0, X). Another example follows from the surveillance of the highly pathogenic avian influenza (HPAI) H5N1. Many farmers are reluctant to report H5N1 cases among their livestock because of the considerations of economic loss [59, 60]. Hence, many locations with HPAI H5N1 cases are subsequently misclassified as locations without cases. These results are then linked with spatial location such as residential or farm address for spatial epidemiological studies, where the location-based outcome measurement errors occur. This will bias the results of spatial epidemiologic studies.

Li et al. [61] found that the misclassification of birth defects can elevate the state-wide congenital anomaly reporting rate from 1.1 to 1.8 % of live births, and after removing the misclassified data geographic clustering in congenital anomaly reports disappeared. Bihrmann et al. [62] explored Bayesian logistic regression to adjust the outcome misclassification and concluded that adjustment for misclassification must be included to produce unbiased regression estimates.

#### Missing outcome measurements

Outcome measurements may be completely missing at some locations for various reasons such as non-responses, uncorrected recording errors, and loss of records. Zukovi and Hristopulos [63] attempted to address the problem of missing values estimation on two-dimensional grids by means of spatial classification methods based on spin models. The “spin” variables provide an interval discretization of the process values, and the spatial correlations are captured in terms of interactions between the spins. The spins at the unmeasured locations are classified by means of the “energy matching” principle: the correlation energy of the entire grid (including prediction sites) is estimated from the sample-based correlations. They also compared the spin-based methods with standard classifiers such as the *k*-nearest neighbor, the fuzzy *k*-nearest neighbor, and the support vector machine (SVM), finding that the spin-based classifiers provide competitive choices. While in classical statistics, the techniques such as multiple imputation have been widely studied [64], the extension of these approaches to address missing data in spatial analyses has not received wide attention. Within the framework proposed in this work, the true and unobserved Y would be replaced by Y_0_, estimated via a function-dependent on both spatial and non-spatial terms. In Manjourides et al. [58], the missing outcomes were imputed based on both the distance from the nearest health center and a disease-specific covariate that was not spatially dependent. Assuming the covariates are measured with no errors, non-zero gammas could be present in the estimation of the distance from the nearest health center (nonzero *γ*_0_, *γ*_1_) and in the estimation of the outcome (non zero *γ*_3_, *γ*_4_). If we assume that the location and covariate are both measured without errors, then following the reasoning from multiple imputation, we could set *γ*_4_ equal to the average of the observed Ys times an indicator that is 1 when Y is unobserved and 0 otherwise.

### Location-based covariate measurement errors

Pure covariate location measurement errors occur when imprecise locations are used for covariate measurements. Covariate measurement errors have been commonly observed in public health studies. Li et al. [65] showed that ignoring this type of measurement errors would result in attenuated regression coefficients and in inflated variance components. For spatial covariate measurement errors, the imprecise address proxies are the major issue, while the traditional nonspatial covariate measurement errors has been studied widely and will not be discussed further [66, 67].

Imprecise address proxies occur when a single measure (e.g., central monitor data or spatial average estimates using data from multiple monitors) is used to characterize a covariate such as ambient pollutant levels across a study area [68]. Only under the strong assumption of spatial homogeneity, will the problem of address proxies be avoided.

Few studies have reported and compared the positional discrepancies between address proxies and the exact address they are supposed to represent. Bow et al. [69] determined the locations in meters for both the street address (location of residence) and postal code location for each cardiac catheterization case in an urban Canadian City and found that 87.9 % of the postal code locations were within 200 m of the true address location (straight line distances) and 96.5 % were within 500 m of the address location, suggesting in this case the postal code locations may be a reasonably accurate proxy for address location. However, Healy et al. [70] quantified the magnitude of distance errors and accessibility misclassification that result from several commonly-used address proxies in spatial epidemiologic studies. They found that using address proxies based on large aggregated units such as centroids of census tracts or dissemination areas can result in large positional discrepancies (with median errors of 343 and 2088 m in urban and rural areas, respectively) and the commonly used proxies for residential address such as postal code centroids can also have large positional discrepancies (median errors of 109 and 1363 m in urban and rural areas, respectively), and are prone to misrepresenting accessibility in small towns and rural Canada. Lo Iacono et al. [71] found that mapping owner addresses as a proxy for horse location significantly underestimates the risk of an outbreak of African horse sickness (AHS) in Great Britain. Duncan et al. [72] used three different “neighborhood” definition, including specific home addresses, census block groups, and census tracts, to explore how neighborhood definition influences the measurement of youths’ spatial accessibility to tobacco retailers and found that measurements of neighborhood exposures likely vary depending on the definition of “neighborhood” selected. Accordingly, address proxies should be used with caution in spatial epidemiologic researches, which can lead to the associated nonspatial covariate measurement errors and further bias the results.

In the presence of location-based covariate measurement errors, we observe the outcome (Y) accurately (*γ*_3_ = *γ*_4_ = *γ*_5_ = 0) and the measured location errors are zero (*γ*_0_ = *γ*_1_ = *γ*_2_ = 0). The errors are completely contained in the covariates which are not accurate (*γ*_6_ ≠ 0, *γ*_7_ ≠ 0, *γ*_8_ ≠ 0). The observed data takes the form (*L*_0_, *Y*_0_, *X*).

### Covariate-Outcome spatially misaligned measurement errors

Covariate-Outcome spatial misaligned measurement errors comes from the process of alignment between covariate and outcome, which is mainly caused by the inconsistent measurement or usage of location data. The outcome and the covariates are often observed at different locations or aggregated over different geographical units. Such data are said to be spatially misaligned. For example, a point-to-point misalignment problem arises when relating air quality measurements, observed at monitors (points), and birth weights observed at the residential locations of the mothers (different points). In many spatial epidemiologic studies, the locations of covariates and health outcomes do not match.

Standard regression methods cannot be applied to such misaligned data. To overcome this problem, several methods have been proposed. Most approaches involve directly using predictions from statistical exposure models that incorporate spatial structure [73–75] such as kriging and its extensions [76], Gaussian process (GP) modeling and Bayesian smoothing [77, 78], penalized regression splines [73, 79], and kernel smoothing [80], among others. The most common strategy is to employ one of the previously mentioned models to predict covariates at locations with outcomes and then estimate a regression parameter of interest using the predicted covariates. For example, Higgins et al. [81] used polynomial regression to generate covariate predictions when outcomes and covariates were misaligned. Waller and Gotway [82] used kriging to predict exposures and used resampling to account for the uncertainty in using the predictions in place of the true values. Kunzli et al. [74] assigned exposure values for subject-specific locations derived from a geostatistical model and used weighted least squares in the subsequent health effects model with the weights specified as the inverse of the standard errors (SEs) from the exposure kriging model. For a similar problem, Madsen et al. [83] considered both a generalized least squares (GLS) estimator with a bootstrap type variance estimator and a maximum likelihood approach that jointly fits the exposure and health models.

Such covariate predictions contain measurement errors since the predicted values will not equal the true exposures [84, 85]. Using predictions rather than true exposures in health modeling introduces two sources of measurement error-*Berkson*-*like errors* arising from smoothing the true covariate surface and *classical*-*like errors* coming from estimating the covariate model parameters, which have been widely discussed in environmental epidemiology [86]. For example, when assessing health effects of particulate matter (PM) constituents, it is necessary to effectively estimate exposure and to account for exposure errors induced by spatial misalignment to avoid bias [87]. After characterizing the spatial misalignment using geostatistical methods, Goldman et al. [88] found that errors due to spatial misalignment resulted in average risk ratio reductions of <16 % for secondary pollutants (O3, PM2.5 sulfate, nitrate and ammonium) and between 43 and 68 % for primary pollutants (NO_x_, NO_2_, SO_2_, CO, PM2.5 elemental carbon) while pollutants of mixed origin (PM10, PM2.5, PM2.5 organic carbon) had intermediate impacts. Sheppard et al. [89] found that measurement errors resulting from spatial misalignment led to an attenuation of acute health effect estimates of 7.7 % for PM2.5 mass. Ong et al. [90] found that relative to geographically corrected data, spatial misaligned information produced a modest bias in the aggregated number of facilities at risk but generated a substantial number of false positives and negatives.

Some methods to correct for spatial misalignment have been proposed. Lopiano et al. [91] developed an approach for an REML-based estimated generalized least squares (RBEGLS) estimator accounting for the misalignment error structure and estimating covariance parameters using likelihood-based methods. They also provide insights into when it is important to fully account for the covariance structure induced from the different error sources. These researchers also developed another pseudo-penalized quasi-likelihood algorithm to account for spatial misaligned errors and showed by simulation that the method performs well in terms of coverage for 95 % confidence intervals [92]. Szpiro et al. [93] characterized the measurement errors by decomposing it into Berkson-like and classical-like components and developed two correction approaches of the parametric bootstrap and the “parameter bootstrap” [86] and also proposed another robust method for the spatially misaligned errors to correct finite-sample bias and correctly estimate standard errors. Gryparis et al. [84] developed a generalized linear model framework for spatial misaligned measurement error modeling; Chang et al. [94] addressed the challenge of exposure measurement errors due to spatial misalignment through measurement error modeling and developed a Bayesian framework to fully account for uncertainty in the estimation of model parameters. Bayesian hierarchical models which account for uncertainties due to spatial misalignment were also applied to spatial correlated exposures measured with errors by Smith et al. [95]. and Molitor et al. [96].

In the presence of Covariate-Outcome spatial misaligned measurement errors, the covariate is not accurate (*γ*_6_ ≠ 0, *γ*_7_ ≠ 0, *γ*_8_ ≠ 0). The mismeasured locations and the outcome (Y) are only affected by themselves (*β*_0_ ≠ 1, *β*_4_ ≠ 1). The observed data takes the form (*L*, *Y*, *X*).

## Discussion

Like measurement errors in classical epidemiology, spatial measurement errors are also ubiquitous in spatial epidemiology. Some types of errors have been widely recognized and extensively studied in other disciplines. For example, geocoding errors arising from non-instrumental measurement errors are widely discussed in the field of geography and concern with spatial misaligned measurement errors have been raised in air pollution studies. However, we are not aware of previous efforts to systematically review and classify the types of spatial measurement errors.

We proposed a classification framework for spatial measurement errors which includes four categories (see summaries in Table 1). We then integrated these with a unified theoretical model; while we illustrated each type of errors as an isolated effect, in practice, many measurement errors can occur simultaneously. For simplicity, we have described only non-differential errors since differential errors (i.e. those errors in the probability of errors differs by location) will cause even greater mischief.

**Table 1:** Summaries of spatial measurement errors

Classes	Subclass	Observed data	Nonzero γs
Pure spatial location measurement errors	Instrumental errors (e.g., global positioning systems errors)	(L, Y_0_, X)	*γ*_0_, *γ*_6_, *γ*_8_
	Non-instrumental errors: multiple address	(L, Y_0_, X)	*γ*_0_, *γ*_6_, *γ*_8_
	Non-instrumental errors: geocoding errors	(L, Y, X)	*γ*_0_, *γ*_2_, *γ*_3_, γ_6_
	Non-instrumental errors: outcome aggregations	(L, Y, X)	*γ*_0_, *γ*_3_, *γ*_4_, γ_8_
	Non-instrumental errors: covariate aggregations	(L, Y_0_, X)	*γ*_0_, *γ*_6_, γ_8_
Location-based outcome measurement errors	Purely outcome measurement errors	(L_0_, Y, X_0_)	γ4
	Missing outcome measurement	(L_0_, Y, X_0_)	γ4
Location-based covariate measurement errors	Location-based covariate measurement errors	(L_0_, Y_0_, X)	*γ*_6_, *γ*_7_, γ_8_
Covariate-Outcome spatial misaligned measurement errors	Covariate-Outcome spatial misaligned measurement errors	(L, Y, X)	*γ*_0_ *, γ*_4_ *, γ*_6_, *γ*_7_, γ_8_

*L* = *L*_0_ + Δ_L_, where Δ_L_ = *γ*_0_L_0_ + *γ*_1_Y + *γ*_2_X + ε_L_; *Y* = *Y*_0_ + *Δ* *Y, where ΔY* = *γ*_3_ *L* + *γ*_4_ *Y*_0_ + *γ*_5_ *X* + ε*y*; *X* = *X*_0_ + Δ_X_, where Δ_X_ = *γ*_6_L + *γ*_7_Y + *γ*_8_X_0_ + ε_X_. (*L*_0_, *Y*_0_, *X*_0_), (*L*, *Y*, *X*) and (Δ_L_, Δ_Y_, Δ_X_) are the true measures, observed values and measurement error of the outcome location, outcome, and covariate measures, respectively

In this effort to review spatial measurement errors, we hope to attract the other spatial epidemiology researchers’ attention to this common challenge. We believe there is opportunity for methodologists to develop new approaches for addressing spatial measurement errors, and provide some useful tools for applied spatial epidemiologists. We know our classification may be not perfect (e.g., geocoding errors can further result in the exposure/covariate measurement errors, suggesting there might be somewhat “overlapping”) [97], but we are confident that this first attempt to add structure to this issue will generate greater discussion, consideration, and solutions to these very practical problems.

## Conclusion

Spatial measurement errors are ubiquitous threat to the validity of spatial epidemiological studies. We propose a systematic framework in this study for understanding the various mechanisms which generate spatial measurement errors and present practical examples of such errors and potential solutions.
